# Development and Evaluation of Scaffolds Based on Perch Collagen–Hydroxyapatite for Advanced Synthetic Bone Substitutes

**DOI:** 10.3390/pharmaceutics18010033

**Published:** 2025-12-26

**Authors:** Alina Elena Coman, Ana Maria Rosca, Maria Minodora Marin, Madalina Georgiana Albu Kaya, Raluca Gabor, Catalina Usurelu, Mihaela Violeta Ghica, Laurentiu Dinca, Irina Titorencu

**Affiliations:** 1 Collagen Department, Division Leather and Footwear Research Institute, National Research and Development Institute for Textiles and Leather, 93 Ion Minulescu St., 031215 Bucharest, Romania; coman.alina27@yahoo.com; 2 Institute of Cellular Biology and Pathology “Nicolae Simionescu”, 8 B. P. Hasdeu Street, District 5, 050568 Bucharest, Romania; ana-maria.rosca@icbp.ro (A.M.R.); irina.titorencu@icbp.ro (I.T.); 3 Advanced Polymer Materials Group, University POLITEHNICA of Bucharest, 1-7 Polizu Street, 011061 Bucharest, Romania; minodora.marin@ymail.com; 4 National Institute for Research & Development in Chemistry and Petrochemistry—ICECHIM, 202 Splaiul Independentei, 060021 Bucharest, Romania; ralucagabor@yahoo.com (R.G.); usurelu_catalina@yahoo.ro (C.U.); 5 Department of Physical and Colloidal Chemistry, Faculty of Pharmacy, Carol Davila University of Medicine and Pharmacy, 050474 Bucharest, Romania; mihaela.ghica@umfcd.ro; 6 Innovative Therapeutic Structures Research and Development Center (InnoTher), Carol Davila University of Medicine and Pharmacy, 050474 Bucharest, Romania; 7 National Research and Development Institute for Textiles and Leather (INCDTP), 030508 Bucharest, Romania; laurentiu.dinca@incdtp.ro

**Keywords:** biopolymer materials, biomaterials, bioavailability, marine collagen, bone defects

## Abstract

**Background/Objectives:** Bone defects remain widespread. Type I collagen–hydroxyapatite composites suit bone engineering by mimicking matrix structure, making them pertinent materials for bone tissue engineering across a range of defect types. Their application is well aligned with non-load-bearing conditions, while use in load-bearing sites requires mechanical properties that meet the demands of those environments. Marine collagen offers a low-cost source from processing by-products. This work aimed to develop perch collagen–hydroxyapatite scaffolds for bone tissue engineering. **Methods:** Composites with COLL:HAp ratios of 100:0, 50:50, 40:60, and 30:70 were prepared. After crosslinking and freeze-drying, porosity and water absorption were examined. SEM and X-EDS assessed morphology and elemental distribution. FT-IR confirmed the chemical composition. Compression tests evaluated mechanical behavior. Cell viability and colonization assessed the biological performance. Biodegradability, thermal stability, and antimicrobial activity were also determined. **Results:** FT-IR confirmed the characteristic absorption bands of both components. SEM and swelling behavior showed porous, interconnected structures with uniform hydroxyapatite dispersion. X-EDS indicated Ca/P ratios consistent with hydroxyapatite. Thermal analysis demonstrated scaffold stability. Compression tests showed mechanical resistance for all the scaffolds, with stiffness increasing with the inorganic content. Perch collagen enhanced biological functionality, supporting osteoblast viability and colonization. Biodegradation gradually proceeded. Antibacterial activity against the tested pathogens was detectable, though moderate. **Conclusions:** The developed scaffolds combined structural stability, controlled degradation, and favorable cell response, constituting a viable and promising candidate for applications in bone tissue engineering.

## 1. Introduction

Bone defects constitute a major global health concern, affecting millions of people [1]. Osteoporosis alone is estimated to affect more than 200 million people worldwide [2,3,4]. The reduction in bone mass and structural integrity leads to increased risk of fracture, thereby diminishing patients’ quality of life and creating significant socioeconomic and healthcare burdens for patients [5]. Bone tissue engineering represents a regenerative medicine approach, designed to restore and reconstruct bone defects through the integration of biomaterials, cells, and growth factors, stimulating the formation of new bones. This multidisciplinary strategy seeks to overcome the inherent limitations (limited availability of donor tissue and the risk of significant postoperative problems) associated with conventional bone grafting procedures by enabling the development of functional bone tissue capable of repairing bone defects caused by trauma, pathological processes, or age-related degeneration [6,7]. Researchers have explored bone tissue engineering and material science to develop replacement materials that can function as graft alternatives [8,9].

Within this context, biomaterials play an essential role in bone tissue engineering, serving as essential components and acting as three-dimensional scaffolds that provide mechanical support and a biologically favorable microenvironment for cellular adhesion, proliferation, and differentiation [10,11]. The biocomposites guide and sustain the regeneration of osseous tissue, performing the role of a temporary matrix in bone repair, while mimicking the physicochemical and structural characteristics of native bones. Their porosity is a key design parameter, facilitating cellular infiltration, nutrient exchange, and vascularization, factors crucial for effective tissue integration and maturation [3,12].

Effective biomaterials must possess biocompatibility, controlled biodegradability, and increased bioactivity, enabling dynamic interaction with host tissues to accelerate healing processes. The development of biomaterials progresses from basic biocompatible materials to advanced, bioactive materials that can be tailored to specific requirements. Biomaterials promote osteoconduction (directing bone growth) and, in certain situations, osteoinduction (inducing bone formation directly) [13,14].

Over the past years, multiple bone substitute materials have been designed and developed, with some having progressed to clinical application, including metals and alloys [15], minerals such as hydroxyapatite, tricalcium phosphate, and other calcium phosphate compounds [16], synthetic polymers [17], naturally derived polymers [18], and a wide range of composite materials integrating these components.

It is well established that bone is composed mainly of an inorganic phase, accounting for approximately 70% of its total mass, primarily consisting of hydroxyapatite (HAp) crystals. The predominant organic component is type I collagen, which represents about 30% of the bone composition [19]. Together, these two components form the primary structural framework of bone. In addition to collagen and HAp crystals, bones also contain non-collagenous proteins, lipids, and water. The mineral component of bone, HAp, is a calcium phosphate mineral with the composition [Ca_5_(PO_4_)_3_(OH)] that crystallizes on collagen fibers, depositing and hardening the tissue to give bone its strength and stiffness [20]. Collagen, the primary organic component, forms a matrix that supplies flexibility and mechanical support while also promoting cell adhesion, migration, and proliferation, processes essential to bone growth and repair. Extensive research on collagen–HAp composites support several bone graft substitutes because these materials replicate both the structural framework and the cellular environment required for new bone formation [21,22].

Representative examples include the development of marine collagen/silica biocomposites for tissue engineering applications [23], multifunctional curcumin-loaded graphene oxide–incorporated collagen scaffolds designed for bone regeneration [24], and bioresorbable purified fibrillar collagen combined with HAp/β-tricalcium phosphate ceramic composites employed as bone substitute materials for socket preservation after tooth extraction [25]. Collagen–HAp composites have been widely reported using mammalian and non-mammalian collagen sources. Representative studies include bovine Coll–HAp scaffolds showing improved mechanical stiffness and osteoconductivity [26], porcine collagen matrices with HAp coatings for periodontal/bone applications [27], and marine-derived teleost collagens combined with mineral or polymeric phases to produce porous 3D scaffolds [28]. Recombinant human collagen (RHC) has also been used recently to fabricate porous scaffolds as a non-animal alternative [29].

Recent studies have increasingly focused on marine collagen, highlighting its potential as a superior alternative to mammalian sources. Compared with collagen derived from mammals, marine collagen provides multiple benefits, including low immunogenicity, lack of zoonotic diseases, ease of absorption, biocompatibility, and lower environmental contamination. Furthermore, marine-derived collagen is an ideal product because it raises no ethical or religious issues. Additionally, marine collagens are more affordable than collagen derived from mammals, being primarily extracted from industrial waste and by-products [23,30,31,32].

Based on this data, the present work focused on developing novel scaffolds based on fish-derived collagen and HAp, with potential application as advanced synthetic bone substitutes in bone tissue engineering, starting from natural resources such as fish skin. The marine collagen extraction process with ascorbic acid from European perch skin (*P. fluviatilis*) and characterization were performed in our previous study [33]. The innovative aspect of this research lies in the fact that to our knowledge there are no published reports specifically combining European perch collagen with HAp to form 3D porous scaffolds. This represents a knowledge gap which our study addresses. Furthermore, the rationale for selecting *P. fluviatilis* lies in its substantial consumption across Europe, which generates considerable volumes of processing by-products.

However, there is a study in the literature that analyzes the quantitative properties of the collagen extracted from European perch skin, supporting its suitability for further uses: hydroxyproline approximately 83 residues per 1000 (estimated), total imino acids approximately 192 residues/1000, a gel strength of approximately 184 g, a melting point of approximately 24.5 °C, and an extraction yield of 10.2–13.8% (based on wet weight) [34]. Marine collagen shows, in general, lower zoonotic/viral risk, antibacterial properties, and reduced mammalian antigenicity compared with bovine/porcine collagen, and they enable the valorization of waste streams. These numeric metrics (hydroxyproline/imino acid content, gel strength, yield) are useful key factors when discussing stability, thermal properties, and crosslinking strategies for bone scaffold fabrication.

It is well known that marine collagen can release antibacterial peptides such as collagencin, which adopt β-sheet or β-turn conformations and disrupt bacterial membranes through a carpet-like mechanism [35]. This activity has been confirmed for peptides isolated from fish collagen. HAp further influences bacterial behavior by altering the scaffold’s surface topography, roughness, and hydrophilicity, changes that can reduce the initial adhesion of the bacteria. HAp crystals may also affect the local environment through calcium ion interactions, contributing to lower bacterial colonization [36].

In scaffolds consisting of mixing marine collagen and hydroxyapatite, peptides derived from collagen provide direct antibacterial action, while HAp particles limit bacteria adhesion. The result is a complementary, dual mode of protection that preserves the regenerative potential of collagen–HAp composites.

Starting from the bone composition mentioned above, the objective of this research was to continue a previously published study and develop collagen–HAp scaffolds, biomaterials proposed for bone regeneration. The 50–70 wt.% HAp range reported in the manuscript was chosen following preliminary formulation and processing assessments. Compositions containing less than 50 wt.% HAp exhibited marked phase separation and inadequate mineral dispersion under the employed fabrication conditions, which prevented their reliable evaluation and comparison. The maximum of 70 wt.% HAp was chosen according to the bone composition. Structural analysis via FT-IR confirmed the functional groups, highlighting the main characteristic absorption bands corresponding to the organic and inorganic phases, while SEM analysis revealed a uniform dispersion of the ceramic particles and suitable porosity for bone in-growth. The porous structure of collagen scaffolds was also demonstrated by their swelling behavior. The use of marine collagen, extracted from perch skin, enhanced the biological functionality of the scaffold, leading to increased osteoblast viability and potential antibacterial properties against *Escherichia coli* (*E. coli*), *Staphylococcus aureus* (*S. aureus*), and *Pseudomonas aeruginosa* (*P. aeruginosa*). The biodegradability over time and the thermal properties of the scaffolds were also investigated. The findings from the conducted study suggest that the proposed scaffold is a viable candidate for bone regeneration applications. The perch-derived collagen/HA scaffold is conceived for applications in non-load-bearing or low-load-bearing bone defects, such as alveolar bone regeneration, maxillofacial reconstructive procedures, or small metaphyseal voids, where osteoconductivity and gradual biodegradation are desirable.

Collagen–HAp composite scaffolds have been widely studied in dental–alveolar and craniofacial applications, exploiting the osteoconductive and biocompatible nature of HAp/collagen scaffolds [37].

Therefore, the aim of this paper is to extend a previous study by using a new type of marine collagen, extracted from *P. fluviatilis* skin, and develop collagen–HAp scaffolds as biomaterials for bone regeneration, while also valorizing a material normally discarded by the fish-processing industry.

## 2. Materials and Methods

### 2.1. Materials

Type I perch collagen was extracted from fresh perch skin according to the method established in our previous study [33]. Briefly, the method of collagen extraction from perch skin consisted pf maintaining the perch skin for 48 h in the fridge in an acidic medium (in L (+) ascorbic acid purchased from Scharlau, Sentmenat, Spain), followed by 48 h in the fridge in an alkaline medium in the presence of 0.1 M sodium hydroxide, bought from Chemical Company, Iasi, Romania. The skin was then completely dissolved in an ascorbic acid solution. Each step was followed by rinsing the skin with distilled water. Skin pigment was removed with a 2% H_2_O_2_ solution prepared from a 30% hydrogen peroxide stock, purchased from Silal Trading, Bucharest, Romania. All reagents were used as received, without further purification.

### 2.2. Methods

#### 2.2.1. Preparation of Perch Collagen–HAp Scaffolds

The initial perch collagen sample, COLL_P (1.90% in concentration and acidic pH), was diluted to 1% (*w*/*v*) concentration and brought to a pH of 7.2–7.4 using a solution of 1 M sodium hydroxide (Chemical Company, Iasi, Romania) and distilled water under mechanical stirring. The collagen gel yield resulting from the ascorbic acid extraction was 9.2% and the purity of the isolated collagen was demonstrated by the ash content, which was undetectable and reported in our previous study [33]. Then, the HAp powder, (reagent grade, Sigma-Aldrich, Darmstadt, Germany) was added to the diluted COLL_P samples. The perch collagen and HAp powder were mixed in various proportions, as shown in Table 1. Afterwards, the samples were crosslinked with glutaraldehyde, GA (25% aqueous solution, ≥98% purity, provided by Merck, Darmstadt, Germany) and poured into Petri glass dishes.

In order to obtain porous matrices of COLL_P samples, a Delta LSC 2-24 freeze-dryer (Martin Christ, Osterode am Harz, Germany) was used to dry the samples according to a lyophilization program of 48 h.

**Figure 1:**
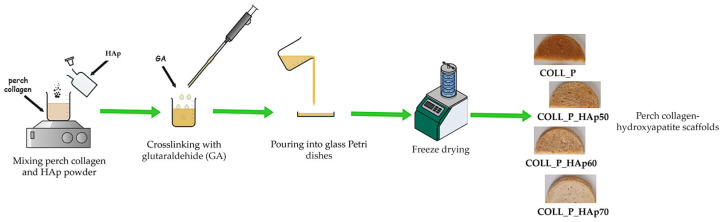
Schematic representation of the scaffold development process based on perch collagen and HAp.

Figure 1 presents the development process of scaffolds based on perch collagen and HAp.

#### 2.2.2. Fourier Transform Infrared Spectroscopy (FT-IR) of Collagen-HAp Scaffolds

To investigate the chemical bonding structure within the perch collagen–HAp scaffolds, the ATR-FTIR technique was employed, using a Bruker Vertex 70 spectrometer (Billerica, MA, USA) equipped with an attenuated total reflectance (ATR) accessory. For each sample, FTIR spectra were acquired in the ATR mode, with a resolution of 4 cm^−1^, across the spectral range of 400–4000 cm^−1^.

#### 2.2.3. Water Absorption

Collagen–HAp scaffolds’ water absorption was determined using the weight method [38]. Initially, a piece of dry porous matrix of about 1 cm^3^ was weighed (*W_d_*). After weighing, the dry samples were placed in 3 mL of distilled water and kept at room temperature for 48 h. At set intervals, the swollen samples were weighed (*W_s_*), and the water retention capacity was determined using Equation (1). The values obtained for water absorption are reported as the mean (±standard deviation), resulting from experiments performed in triplicate.

(1) $$Water\;absorption\;\left(g/g\right)=\frac{W_{s}-W_{d}}{W_{d}}\;\;\;$$

#### 2.2.4. Scanning Electron Microscopy (SEM) of Collagen-HAp Scaffolds and X-Ray Energy-Dispersive Spectrometry (X-EDS)

The scanning electron microscopy of COLL_P samples was performed with FEI Quanta 200 (FEI Company, Hillsboro, OR, USA) using a tungsten filament at the acceleration voltage of 10 kV and the emission current in the range of 95 ÷ 105 µA. The 2D morphological images were acquired with an LFD (Large Field Detector) for secondary and back-scattered electrons in low-vacuum conditions (130 Pa pressure) with water vapors. This vacuum mode allowed for electric charge compensation on the sample surface by the water molecules, thus avoiding sample metallization, which would have been necessary in high-vacuum conditions. The samples were fixed on a metallic stub using a double-sided conductive-carbon adhesive tape for the ground connection of the sample.

The pore diameter of the prepared sponges was measured from at least five SEM micrographs using the ImageJ software (Version 1.8.0). For each type of sample, about 185 pores were analyzed to obtain the pore size distribution.

The samples’ porosity was determined using the Adjust Threshold tool in the ImageJ 1.8.0 software and computed as the ratio between the cumulative area of the pores and the total area of the examined surface, based on the SEM images recorded at 200× magnification.

The X-ray energy-dispersive spectrometry (X-EDS) was performed with a EDAX Ametek Element system. This analysis allows for the identification of chemical elements at sample’s surface by X-ray fluorescent emission after the electron beam interaction. The used acceleration voltage was set to 10 kV; therefore, the maximal emitted spectral energy was 10 keV.

#### 2.2.5. Mechanical Properties of Collagen-HAp Scaffolds

Compressive tests of the samples were carried out on a DMAQ800 (TA Instruments, New Castle, DE, USA). The measurement method was DMA Controlled force, mode Stress/Strain, isotherm at 25 °C, using penetration clamp on cylindrical samples with dimensions of 0.8–12 × 5 mm (length × diameter). All scaffolds were compressed with a ramp force of 0.1 N/min, from 0.1 to 4 N.

#### 2.2.6. Enzymatic Degradation

To investigate the enzymatic degradation of the scaffolds based on perch collagen and HAp, hydrated small pieces of porous matrices resulting from freeze-drying (*W_i_*, initial mass) were immersed in 3 mL collagenase solution of 1 μg/mL (Sigma Aldrich, Darmstadt, Germany) and incubated at 37 °C, the temperature that simulates physiological conditions, monitoring the samples’ degradation over 96 h. At regular time intervals (2 h, 4 h, 6 h, 24 h, 48 h, 96 h), the swollen structures were weighed (*W_t_*), the mass at time *t*. The weight loss of the samples was calculated according to Equation (2). For all the samples, each measurement was performed in triplicate, and the results represent the mean ± standard deviation.

(2) $$Weight\;loss\;\left(\%\right)=\frac{W_{i}-W_{t}}{W_{i}}\times 100\;\;$$

#### 2.2.7. Thermogravimetric Analysis (TGA) of Collagen–HAp Scaffolds

The thermal behavior of perch collagen–HAp samples was investigated using an NETZSCH TG 209 F1 Libra instrument (NETZSCH-Gerätebau GmbH, Selb, Germany) under a controlled nitrogen atmosphere with a flow rate of approximately 20 mL/min. The samples were heated from 25 to 700 °C at a constant rate of 10 °C/min. Each measurement was performed on sample with weights ranging between 5 and 6 mg, in triplicate.

#### 2.2.8. Antimicrobial Activity of Collagen–HAp Scaffolds

The antimicrobial assay was carried out via a modified disk diffusion method using reference strains from the Microbiology Department of the INCDTP collection: *Escherichia coli*, *E. coli*—ATCC 11229, *Staphylococcus aureus*, *S.aureus*—ATCC 6538, and *Pseudomonas aeruginosa*, *P. aeruginosa*—ATCC 27853. Test tubes with Mueller–Hinton agar were inoculated with each strain and incubated for 24 h at 37 °C. Bacterial suspensions of 1.5 × 10^8^ CFU/mL, matching a 0.5 McFarland standard, were prepared from cultures grown on Mueller–Hinton agar. A total of 200 μL of each suspension was spread onto the plates, and COLL_P samples were placed on the inoculated medium. The plates were kept at room temperature to allow for uniform diffusion, then incubated for 24 h at 37 °C. Antibacterial activity of the perch collagen–HAp scaffolds were assessed by the appearance of inhibition zones around the samples. The inhibition zone diameter was measured and calculated using Equation (3):

(3) $$H\;\left(mm\right)=\frac{D-d}{2}\;\;\;$$

where *H* represents the inhibition zone diameter, in mm, *D* is the total diameter of the disk and the area of inhibition, in mm, and *d* represents the diameter of the disk, in mm.

The antimicrobial assay was performed in triplicate. For every sample, the mean and the standard deviation were calculated.

**Figure 2:**
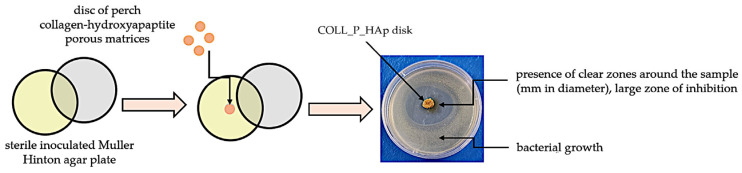
Illustration of the disk diffusion protocol to assess COLL_P samples’ antimicrobial activity.

Figure 2 represents the agar spot diffusion-based screening of COLL_P samples’ antimicrobial activity.

The total aerobic microbial and fungal count of the COLL_P samples was measured to assess contamination. The total aerobic microbial count (TAMC) and the total yeast and mold count (TYMC) assays were conducted using Casein Soya Bean Digest Agar from Novachim for the aerobic count and Sabouraud Dextrose Agar from the same supplier for the fungal count. Plates were incubated at 30–35 °C for one to two days for TAMC and at 20–25 °C for three to five days for TYMC.

#### 2.2.9. Statistical Analysis

Statistical analysis was performed using GraphPad Prism 10.4.2 software. Experimental data were expressed as mean ± standard deviation (*n* = 3 for water absorption, enzymatic degradation studies, and for microbiological evaluation). To analyze differences between groups, two-way analysis of variance (ANOVA) with Dunnett’s multiple comparison test was used. Results were considered significant for *p* < 0.05.

#### 2.2.10. Biocompatibility Evaluation

To assess the biocompatibility of the scaffolds and their capacity to support colonization, we used MG63 (CRL-1427, ATCC, Manassas, VA, USA), a human osteosarcoma cell line suitable for evaluating the interaction with biomaterials designed for bone regeneration. The cells were maintained in low-glucose Dulbecco’s Modified Eagle Medium (Sigma Aldrich, St. Louis, MO, USA) supplemented with 10% fetal bovine serum (Gibco BRL, Gaithersburg, MD, USA) and antibiotics consisting of 100 IU/mL penicillin, 100 µg/mL streptomycin, and 50 µg/mL neomycin (Sigma Aldrich, St. Louis, MO, USA). To evaluate potential dose-related cytotoxic effects, extracts of each scaffold were prepared by incubating the materials in complete culture medium, at a concentration of 0.01 g/mL, for 6 h/37 °C under stirring. Subsequently, the samples were centrifuged for 5 min at 300× *g* and the supernatants were sterilized by filtration (0.2 μm pore size). Serial dilutions were prepared—10 mg/mL, 5 mg/mL, 2.5 mg/mL, and 1.25 mg/mL—and cells were incubated in the presence of extracts for 72 h, when the viability was assessed by XTT assay.

Next, the capacity of the scaffolds to support the growth and colonization with MG63 cells was tested. For this, the materials were cropped with a 4 mm in diameter punch, then the obtained pieces were sterilized by incubation in 70% ethanol overnight, under stirring. The ethanol was removed by washing three times with sterile water and then the cells were maintained in DMEM without serum for at least 24 h. Subsequently, 5 × 10^4^ cells were seeded onto the cropped scaffolds and sustained in culture for up to 4 weeks. One week after seeding, the viability of the cells was measured by XTT assay (Thermo Fisher Scientific, Waltham, MA, USA). For this purpose, the scaffolds were washed with PBS and incubated with 150 µL/well XTT working solution following the producer’s instructions for 3 h at 37 °C, 5% CO_2_. The absorbance was read at 450 nm versus 690 nm using a TECAN spectrophotometer (TECAN, Männedorf, Switzerland). The results were expressed as a percentage of the COLL_P control. Data were analyzed using one-way Anova and are presented as mean ± standard deviation (SD).

To assess the scaffold colonization with MG63 cells, samples collected after 1 and 4 weeks were fixed in 4% PFA and processed for paraffin embedding. Sections of 5 µm thick were prepared with a Leica microtome (Leica, Wetzlar, Germany) and stained with hematoxylin and eosin using a 1 min hematoxylin incubation, followed by 30 s in eosin Y. The stained sections were mounted in Shandon Consul-Mount (Thermo Fisher Scientific, Waltham, MA, USA) and examined with a Zeiss Observer D1 microscope (Zeiss, Oberkochen, Germany).

## 3. Results and Discussion

This research presents an innovative perch collagen–HAp scaffold for bone tissue engineering, developed from marine collagen, extracted from perch skin according to our previous study [30] and HAp incorporated in the collagen matrix. While HAp crystals (HA) mainly form the inorganic phase (around 70% of bone’s composition), providing bone tissue with strength and stiffness, type I collagen is the primary organic component, accounting for around 30% of bone’s composition, offering flexibility and structural support [31,32]. Starting from this, three materials composed of perch collagen (COLL_P) and HAp were prepared to assess the influence of incorporating different percentages of HAp in the collagen matrix, with potential uses in the engineering of bone tissue. The percentages of COLL–HAp used were 50:50, 40:60, and 30:70, respectively. The fourth sample was the control sample and contained only crosslinked perch collagen.

### 3.1. Structural Analysis of Perch Collagen–HAp Scaffolds—FT-IR Spectra

**Figure 3:**
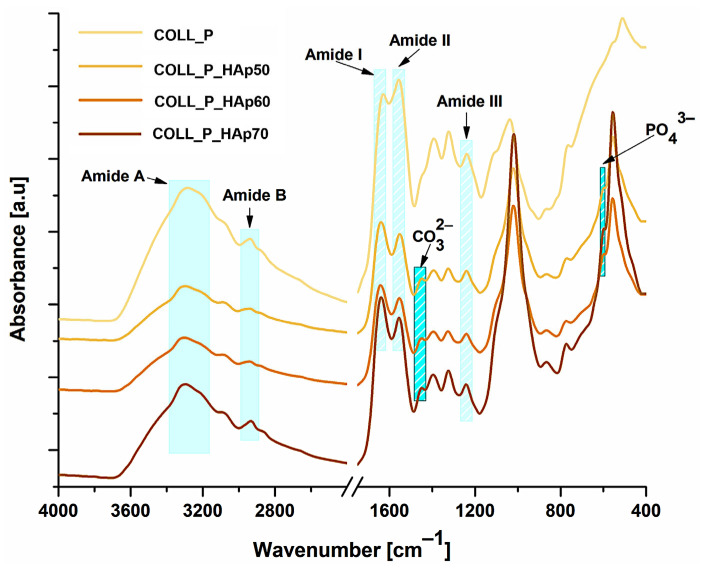
FTIR spectra of perch collagen–HAp scaffolds.

The FT-IR spectroscopy of the obtained scaffolds identifies the functional groups, emphasizing the main characteristic absorption bands corresponding to the organic and inorganic phases. Figure 3 presents the overlapping spectra of COLL_P, COLL_P_HAp50, COLL_P_HAp60, and COLL_P_HAp70 samples.

In all samples, the presence of functional groups characteristic of type I collagen, extracted from perch skin, can be confirmed. The broad band of amide A appears in the range 3295–3305 cm^−1^, mainly associated with the N–H stretch coupled with a hydrogen bond [39]. For the COLL_P sample, the band specific to Amide A presents a shift to a lower frequency, 3295 cm^−1^, compared with samples that contain HAp and present similar Amide A bands: 3301 cm^−1^ for COLL_P_HAp50, and 3305 cm^−1^ for COLL_P_HAp60 and COLL_P_HAp70. This is related to the presence of HAp in the samples. The positions of Amide B bands were found at wavenumbers of 2940 cm^−1^ for COLL_P and COLL_P_HAp50, 2944 cm^−1^ for COLL_P_HAp60, and 2930 cm^−1^ for COLL_P_HAp70. The amide B signal is linked with the symmetrical or asymmetrical stretch of CH_2_ groups. The absorption bands of Amide I were found in the range of 1632–1641 cm^−1^ for the COLL_P samples. The signal may be assigned to the stretching vibration of the C=O groups along the polypeptide structure of collagen. The presence of amide II can be observed for all COLL_P samples at wavenumbers between 1551 and 1556 cm^−1^ and is attributed to NH bending vibration coupled with CN stretching [33,40,41].

Finally, the presence of the amide III signal was identified from the NH bend coupled with CN stretch at peaks in the range 1238–1243 cm^−1^, confirming the characteristic absorption peaks of perch collagen’s secondary structure [42,43].

The FTIR results of perch collagen samples, shown in Figure 3, specifically amide A, B, I, II, and III band vibrations, typical of collagen, are in accordance with other studies based on collagen from marine species [41] and with our previous study, where we extracted collagen type I from perch skin with various acids [33].

The bands identified as amide A and amide I, as mentioned before, are specific to protein structures. In the case of crosslinking, the intensity of the amide I band increases, while that of the amide A band decreases. To determine the samples’ crosslinking grade, the FT-IR absorption ratio of Amide I to Amide A band (A_I_/A_A_) was calculated [44,45]. The Amide I/Amide A ratio reveals the crosslinking degree of perch collagen–HAp scaffolds. The highest degree was observed for the COLL_P_HAp70 scaffold, with an A_I_/A_A_ ratio of 2.36, whereas for the COLL_P_HAp60 scaffold it was 1.46, for the COLL_P_HAp50 scaffold it was 1.38, and it was 1.34 for COLL_P. These results indicate that the crosslinking grade increases with the concentration of HAp in the perch collagen scaffolds.

The bands visible at 1451–1453 cm^−1^ correspond to the carbonate group in HAp and are evident in the samples containing the inorganic phase, while the signal for phosphate is responsible for the bands at 600–602 cm^−1^, confirming the presence of HAp in COLL_P samples.

The spectra follow the expected trend for collagen–apatite composites. The collagen reference (COLL_P) shows only the amide bands, whereas the mineralized samples display the split feature at about 870 and 840 cm^−1^, which is characteristic of B-type carbonate occupying phosphate sites in the apatite lattice. During precipitation, the air exposure allows CO_2_ to dissolve into the reaction medium, where it forms bicarbonate and carbonate. These anions remain available during nucleation and are readily incorporated into the growing crystals. HAp tolerates this substitution well, especially when apatite crystals are small and defective, a common situation in biomimetic systems, unless it is performed under CO_2_-free conditions.

Collagen does not supply carbonate, yet it affects mineral formation. Its functional groups coordinate calcium and phosphate, induce slow crystal growth, and stabilize crystalline apatite domains. This favors lattice configurations in which phosphate positions can be partly replaced by carbonate that is already present in solution. As a result, collagen-mediated mineralization often incorporates more carbonate than purely inorganic precipitation under comparable conditions.

In the COLL scaffolds, the collagen control sample lacks the carbonate band, while the mineralized series shows the doublet, whose intensity grows with increasing mineral content (50, 60, 70). This reflects the larger amount of apatite available to host CO_3_^2−^. The carbonate therefore originates from dissolved atmospheric CO_2_, and collagen enhances its uptake by modifying the crystallization environment rather than generating carbonate itself [46,47,48].

The absorption bands of perch collagen and HAp overlapped in the FT-IR region from 3200 to 3600 cm^−1^, where the OH stretching band associated with the HAp structure and the amide A stretching from collagen are located [49].

### 3.2. Water Absorption of Perch Collagen–HAp Scaffolds

The ability to absorb water represents an important feature of collagen scaffolds intended for tissue engineering use. Adequate values for water uptake enhance the diffusion of water and nutrients into the scaffold, creating a microenvironment favorable for cell growth [50,51].

**Figure 4:**
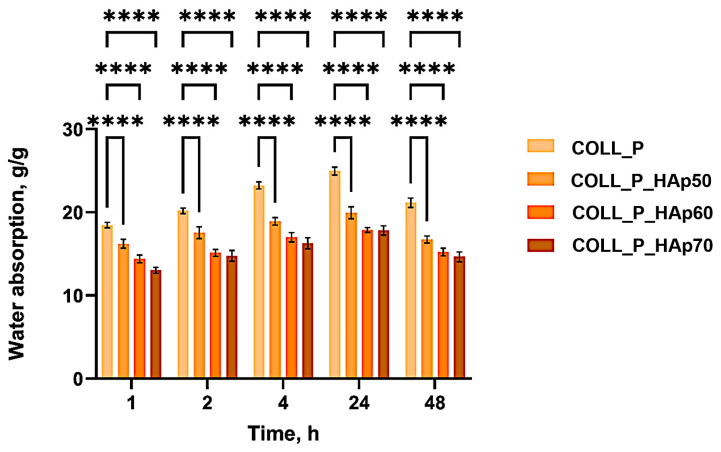
Water absorption ability of perch collagen–HAp scaffolds immersed in distilled water at room temperature for 48 h. Results are statistically significant for *p*-value: **** *p* < 0.0001.

In Figure 4, COLL_P samples’ capacity to swell in distilled water when hydrated for 48 h is presented. The porous structure of perch collagen–HAp scaffolds have a direct impact on the swelling behavior of the samples. The samples absorb between ≈18 and 25 g/g of water. It is well known that marine collagen exhibits a lower swelling degree [13,14], compared with collagen extracted from mammalian species [51].

In the first hours after immersion in distilled water, the samples absorb slowly important amounts of water, reaching equilibrium after one day. COLL_P scaffold swelled the most, absorbing 25 g/g of water after 24 h. After reaching the equilibrium COLL_P sample starts to degrade, and the sample’s weight starts to decrease. Scaffolds with HAp in different concentrations present different swelling behavior. COLL_P_HAp samples absorb lower amounts of water due to the increased density of the samples when adding HAp. As expected, the most compact sample seems to be COLL_P_HAp70 with the highest amount of HAp, which absorbs ≈ 18 g/g of water before reaching equilibrium. After 48 h, the scaffold started to degrade slowly. The same pattern is observed for the COLL_P_HAp60 sample.

COLL_P_HAp50 possesses more pores than the other two, leading to a water uptake of 20 g/g in 24 h. The porous structure of the scaffolds is demonstrated by the scanning electron microscopy images presented below.

Two-way ANOVA with Dunnett’s post hoc test confirmed statistically significant differences between all three samples containing different concentrations of HAp and the control sample COLL_P (**** *p* < 0.0001). These finding highlights the superior swelling performance of COLL_P_HAp50—COLL_P_HAp70 when compared to COLL_P for all time intervals, which could be related to the presence of HAp.

### 3.3. Morphological Analysis of Perch Collagen–HAp Scaffolds

Scanning electron microscopy (SEM) was used to examine the microstructure of the developed scaffolds. Collagen scaffolds’ ability to serve biomedical applications is directly impacted by their porous structure. Cell nutrition, proliferation, and migration for tissue vascularization and the development of new tissues depend on open, porous, and linked structures [52,53]. Furthermore, a porous surface contributes to controlling and promoting the growth of the new tissue [54,55].

SEM analysis revealed that the perch collagen matrix and the HAp granules are uniformly distributed in the COLL_P_HAp samples.

**Figure 5:**
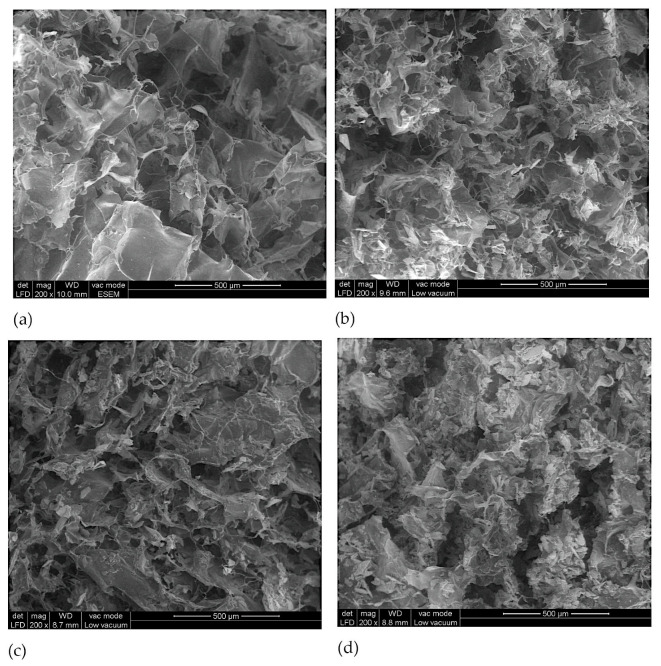
SEM images of perch collagen-HAp scaffolds (magnification 200×): (**a**) COLL_P, (**b**) COLL_P_HAp50, (**c**) COLL_P_HAp60, and (**d**) COLL_P_HAp70.

According to Figure 5, all scaffolds possess an arrangement with irregular interconnected pores, suitable for cell growth and migration, with pore sizes varying from ≈40 μm to ≈150 μm, sizes frequently used in bone tissue engineering [56,57]. There are significant changes in the scaffolds’ structure due to the addition of HAp: the pore distribution decreased in the presence of HAp, as shown in Figure 5b–d, compared with the COLL_P sample shown in Figure 5a, where only the perch collagen crosslinked with GA is presented.

**Figure 6:**
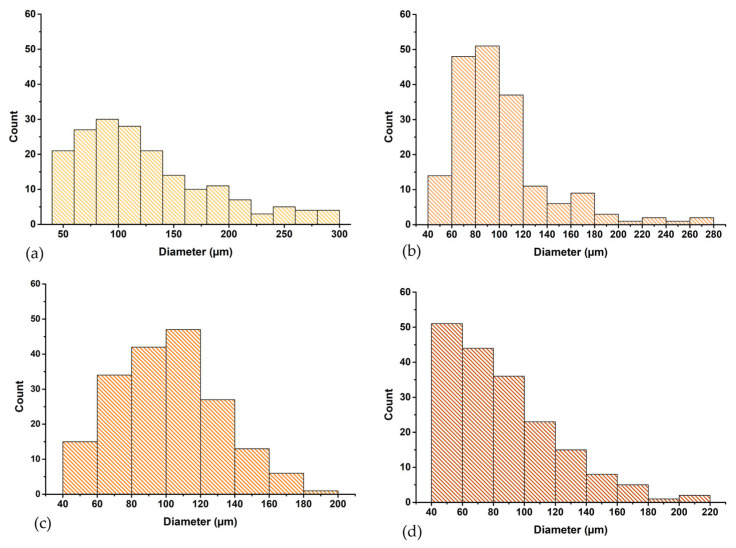
Pore size distribution of COLL_P samples: (**a**) COLL_P, (**b**) COLL_P_HAp50, (**c**) COLL_P_HAp60, and (**d**) COLL_P_HAp70.

Figure 6 compares the pore size distribution of the samples, with the pore sizes of the COLL_P being higher than those of COLL_P_HAp samples, having pore sizes in the range 50–150 μm. The addition of HAp to the COLL_HAp scaffolds resulted in a slight reduction in pore size, and a more compact structure, due to the increased density of the sample. COLL_P_HAp scaffolds feature pores of comparable size and similar porous architecture. In general, COLL_ P_HAp50 presents pore sizes ranging from 60 to 120 μm, while COLL_ P_HAp60 presents pores with dimensions between 60 and 140 μm and COLL_P_HAp70 exhibit pores with sizes between 40 and 120 μm.

Therefore, HAp concentration directly influences the pore size and the structure of the scaffold, with the densest scaffold being COLL_P_HAp70, with the highest quantity of HAp in the composition. The results obtained are in accordance with the swelling behavior of the samples presented in Figure 4.

In addition, the morphological analysis of the perch collagen–HAp scaffolds show the presence of Na element detected by X-EDS, uniformly distributed across the surface, which may originate from the alkaline solution used to decrease gel concentration and increase pH.

In conclusion, a porous fibrous collagen matrix embedded with relatively large HAp particles was revealed by the microstructure of the perch collagen–HAp scaffolds resulting from the SEM analysis.

**Figure 7:**
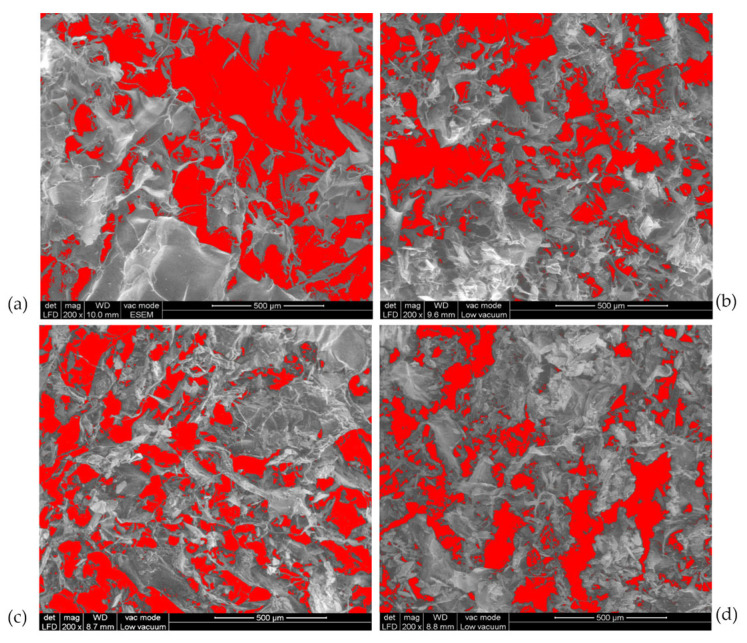
Collagen–HAp scaffold porosity: (**a**) COLL_P, (**b**) COLL_P_HAp50, (**c**) COLL_P_HAp60, and (**d**) COLL_P_HAp70.

The porosity of the samples decreased with the content of HAp. As seen in Figure 7, COLL_P presents a porosity of 37.4%, while for the scaffolds with HAp, the porosity decreased to 25.2% for COLL_P_HAp50, 24.7% for COLL_P_HAp60, and 21.5% for COLL_P_HAp70, respectively.

**Figure 8:**
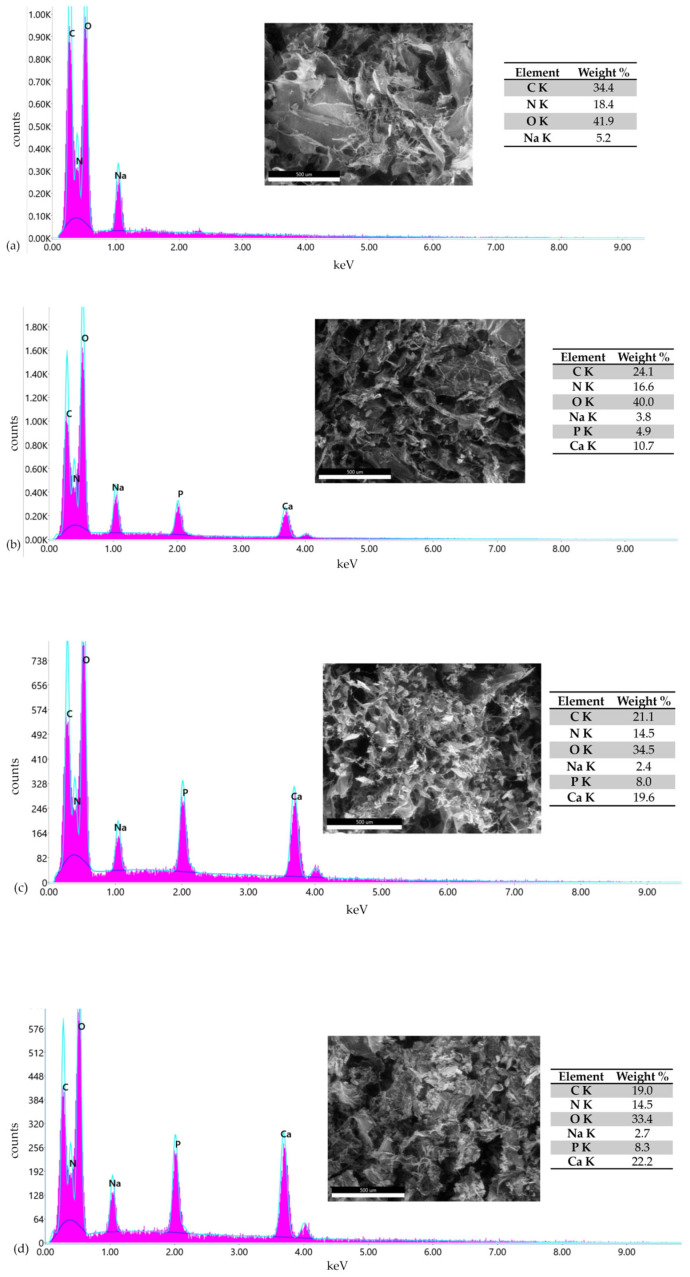
X-EDS analysis for perch collagen–HAp scaffolds: (**a**) COLL_P, (**b**) COLL_P_HAp50, (**c**) COLL_P_HAp60, and (**d**) COLL_P_HAp70.

Energy-dispersive X-ray spectroscopy was used to determine the Ca/P ratio of the scaffolds with collagen and HAp. The amount of Ca and P, as well as the Ca/P mass ratio, are presented. Moreover, the total Ca–P content provides information about the total inorganic fraction of the scaffold. Elemental composition, shown in Figure 8, indicates that the surface contains mainly O (42–33%) and C (19–34%), consistent with collagen functional groups.

Ca (11–22%), P (5–8%) and Na (2–5%) suggest calcium phosphates associated with hydroxyapatite, or, in the case of Na, the element can also originate from the alkaline solution used for pH adjustment and gel extraction. Sodium was also detected in the SEM analysis. In the tables associated with each figure, the elements found on the scaffolds are reported. The results are similar to those in the literature and confirm the presence of hydroxyapatite in the scaffolds [58,59].

X-EDS data indicate that the Ca / P ratios of the analyzed scaffolds exceed the stoichiometric value of hydroxyapatite, 1.67. The measured ratios were 2.18 for COLL_P_HAp50, 2.45 for COLL_P_HAp60, and 2.68 for COLL_P_HAp70, increasing with the HAp content. Elevated Ca / P values are associated with the scaffold’s capacity for biomineralization. Calcium and phosphorus were detected in all collagen–HAp samples.

### 3.4. Compressive Properties of Collagen–HAp Scaffolds

Scaffolds enriched with HAp present a clear reduction in porosity, as discussed previously, once the mineral percentage increases. At the same time, the compression test indicates a notable increase in stiffness and mechanical resistance for samples with HAp, reflecting the role of filler, which compacts the collagen network and consolidates the samples’ structure [60]. This behavior is in accordance with studies from the literature, where the addition of HAp to a collagenic matrix improves mechanical properties, even if the result is low porosity and reduced pores [61,62].

**Figure 9:**
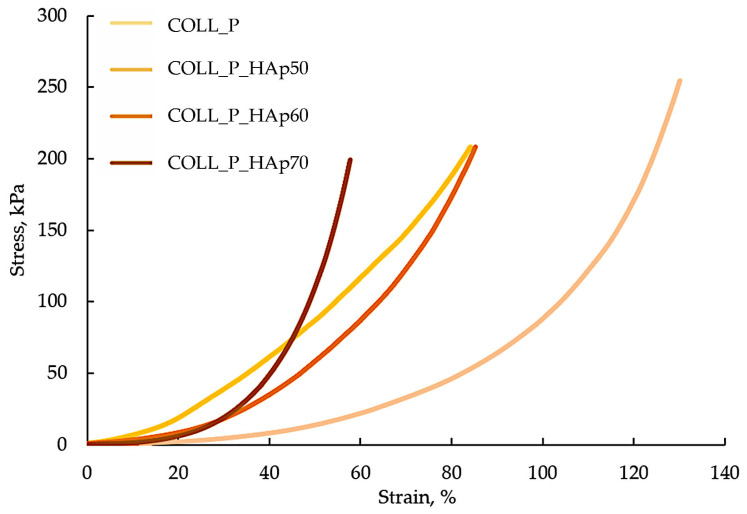
Stress–strain curve for collagen–HAp scaffolds.

The tested sample with an intermediary content of HAp (50–60%) presents an optimal compromise, as seen in Figure 9, combining rigidity and mechanical resistance superior to pure collagen and a pore interconnectivity that likely allows for for cellular infiltration, nutrient diffusion, and vascularization.

As other researchers proved, these type of scaffolds confer a balance between mechanical strength and tissue regeneration facilitation [60,63].

COLL_P_HAp70, provides maximum stiffness, but offers limited deep cellular access and biological remodeling capacity due to its low porosity. In the context of bone reconstruction, the selection of the scaffold should be guided by the specific mechanical and biological requirements: for non-load-bearing or load-bearing defects, moderate variants seem more appropriate; for applications with increased mechanical stress, highly mineralized samples may be suitable, but they must be subjected to an additional evaluation of diffusion, vascularization, and in vivo integration [64].

COLL_P presents the lowest stiffness and rigidity. The stress–strain curve shows low values for stress, suggesting a ductile material favorable for cellular penetration and vascularization, but does not confer enough mechanical resistance.

### 3.5. Enzymatic Degradation of Perch Collagen–HAp Scaffolds

Controlling scaffold degradation is critical for bone tissue engineering. The scaffold must initially resist oxidation in the body fluids in order to promote surface mineralization and secure tissue adhesion, then gradually degrade and support the regeneration of a new uniform bone tissue [65,66]. The cleavage of the peptide bonds from the collagen scaffolds into smaller fragments takes place when collagen meets collagenase. This enzyme plays a significant role in the bone repair process, a process that involves both resorption (breakdown) and formation [66,67].

Modulating collagenase treatment allows for a more precise control of the scaffold degradation to match the tissue regeneration. Localized collagenase application promotes the remodeling of the extracellular matrix, supporting the effective repair of the defects or the damaged tissue. This enzyme specifically recognizes the Pro–X–Gly–Pro sequence from collagen, cleaving the peptide bond between amino acids X and glycine (Gly) [31,68,69].

**Figure 10:**
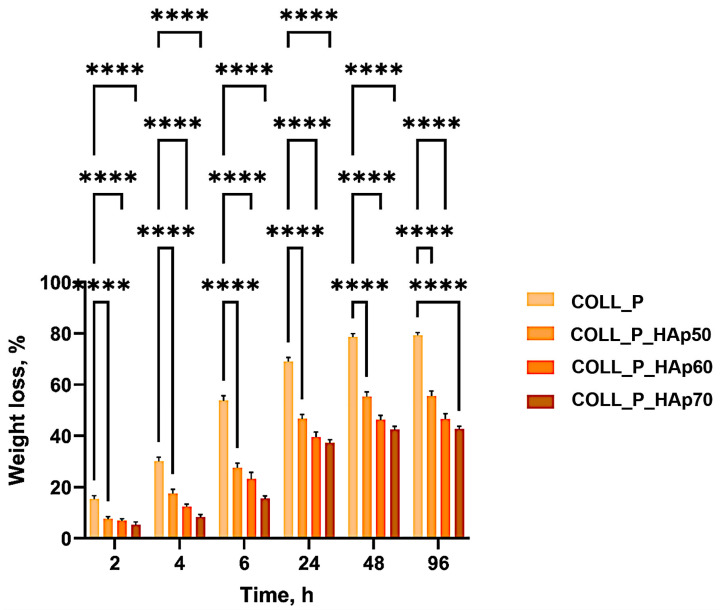
Enzymatic degradation of perch collagen–HAp scaffolds. Results are statistically significant for *p*-value: **** *p* < 0.0001.

Figure 10 illustrates the enzymatic degradation of perch collagen–HAp scaffolds immersed in a 1 μg/mL collagenase solution at 37 °C for 96 h.

The COLL_P_HAp scaffolds present a lower degradation rate compared with the control sample, COLL_P, proving that HAp presents great resistance to collagenase, contributing to the scaffolds’ stability and degradation resistance. After 48 h, samples with HAp maintained their integrity, with a degradation rate of 55% for COLL_P_HAp50, 46% for COLL_P_HAp60, and 42% for COLL_P_HAp70, while the COLL_P sample presented a weight loss of ≈80%. The weight-loss curves approach a clear plateau, and no further degradation is detected beyond 48 h, indicating that the samples remain stable during extended incubation.

The two-way ANOVA combined with Dunnett’s post hoc test revealed an evident statistically significant difference between all three samples containing varying HAp concentrations and the control sample COLL_P (**** *p* < 0.0001), similarly to the water absorption test. These results indicate that COLL_P_HAp50–COLL_P_HAp70 are more stable during collagenase action compared to COLL_P, a finding likely attributable to the presence of HAp.

### 3.6. Perch Collagen–HAp Scaffolds Thermal Analysis

Thermogravimetric analysis was used in this study to demonstrate that mixing perch collagen with HAp can increase the thermal stability of scaffolds. All scaffolds exhibited multi-stage thermal degradation profiles. The initial weight loss observed in the first stage between 25 and 100 °C corresponds to the evaporation of moisture adsorbed by the samples [70]. As can be seen in Table 2, the mass loss is very small, ranging from 2% for COLL_P_HAp70, to 5% for COLL_P.

**Figure 11:**
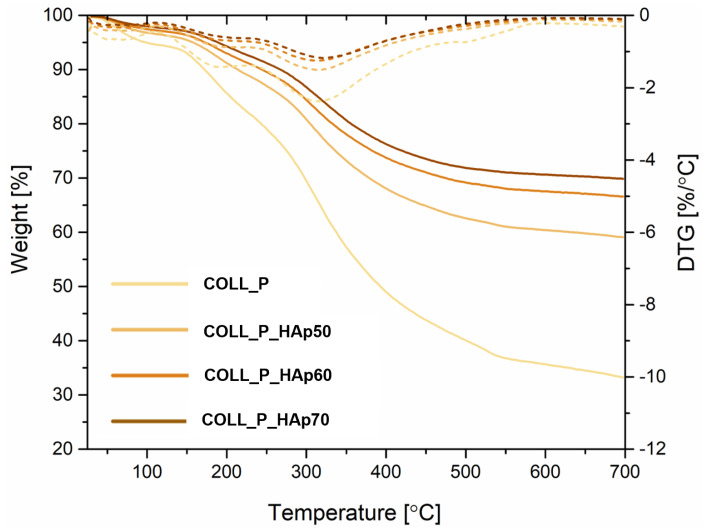
TG and differential TGA (DTG) curves for perch collagen–HAp samples.

According to Figure 11, for all scaffolds, a major degradation stage was observed between 100 and 400 °C, indicating the collagen matrix decomposition and the breakage of peptide; the collagen loses its structural integrity [71,72]. For COLL_HAp samples, one can observe that the weight loss in the second stage is much smaller than for the control sample, COLL_P. The enhanced thermal resistance of COLL_P_HAp scaffolds is supported by their percentage weight loss in the second stage: between 100 °C and 400 °C (28% for COLL_P_HAp50, 23% for COLL_P_HAp60, and 22% for COLL_P_HAp70), comparatively less than for the COLL_P sample (46%). The presence of HAp in the scaffolds, a thermally stable bioceramic, significantly improves the resistance of the scaffolds to thermal degradation, as can be observed in Table 2.

The third stage of scaffolds’ decomposition was related to collagen carbonization, characterized by a small mass loss, from 16% for COLL_P to only 6% for COLL_P_HAp70, leaving a residue at 700 °C of 33% for COLL_P, 59% for COLL_P_HAp50, 67% for COLL_P_HAp60, and 70% for COLL_P_HAp70, corresponding to the calcium phosphate ceramic for COLL_P_HAp samples. The maximum temperature denaturation peak on the DTG curves is quite similar for all the scaffolds, 315 °C for COLL_P, COLL_HAp50 and 60, and 321 °C for COLL_HAp70.

Nevertheless, the COLL_HAp samples showed a lower degree of thermal degradation, confirmed by the higher residual mass, compared with the control sample, COLL_P, proving that the perch collagen–HAp scaffolds developed are highly stable at high temperatures. The thermal results achieved for the perch collagen–HAp scaffolds surpass studies reported earlier that incorporated an inorganic phase into another marine collagen matrix, for example, marine sponge collagen [73] and collagen type I from *Nile tilapia* [74].

### 3.7. Collagen Porous Matrices Microbiological Analysis

Enhancing the antibacterial properties of the biomaterials represents a critical factor in order to achieve an optimal infection control [75]. The antibacterial activity of collagen scaffolds used for bone tissue engineering plays an essential role in preventing and managing bone infections, which remain a major clinical challenge due to their potential to hinder tissue regeneration, cause implant failure, and substantially increase healthcare costs. Therefore, the incorporation of antibacterial properties is imperative. Because microbial infections can severely reduce cell viability and significantly increase the risk of implant failure, scaffolds possessing antimicrobial properties show significantly increased potential for clinical success [76,77]. For this reason, the antibacterial activity of perch collagen–HAp scaffolds were assessed.

**Figure 12:**
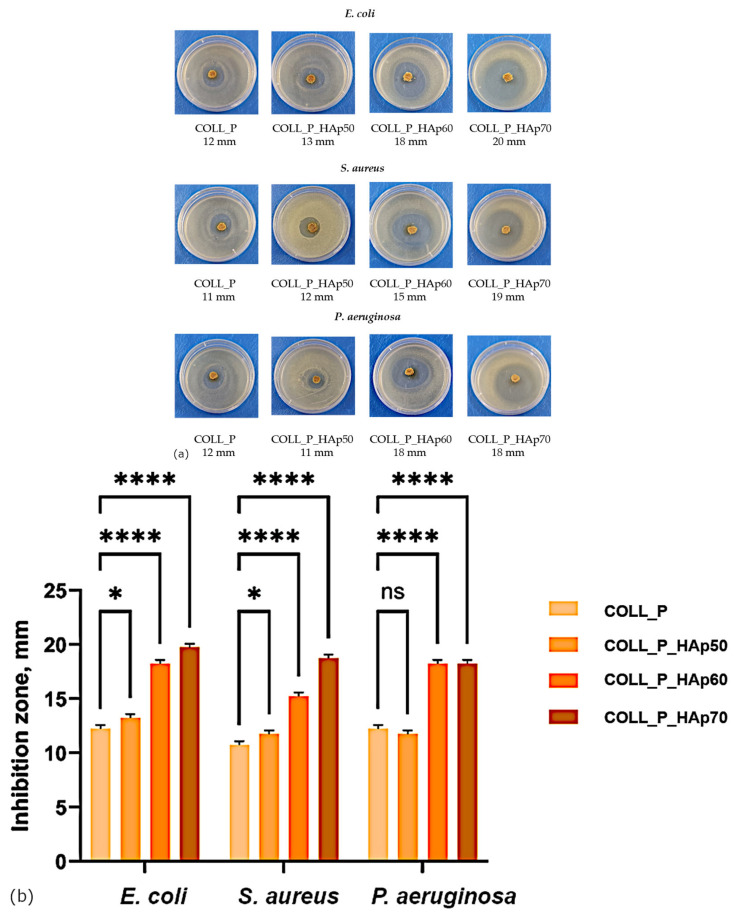
Antibacterial activity of perch collagen–HAp scaffolds against *E. coli*, *S. aureus*, and *P. aeruginosa*, determined by the disk diffusion method: (**a**) digital photos of agar plates with inhibition zones; (**b**) statistical analysis. Results are not statistically significant (ns) for *p*-value > 0.05; results are statistically significant for *p*-value as follows: * *p* < 0.05; **** *p* < 0.0001.

Figure 12 shows the antibacterial activity of COLL scaffolds, highlighting their ability to suppress the growth of common pathogenic bacteria: the Gram-negative strains *E. coli* and *P. aeruginosa*, and the Gram-positive strain *S. aureus*.

All the samples exhibit high antimicrobial activity, demonstrating the efficiency of the perch collagen–HAp scaffolds in bone tissue engineering. The highest inhibition zone for the tested microorganism, *E. coli*, *S. aureus*, and *P. aeruginosa*, was achieved by COLL_P_HAp 70, followed by COLL_P_HAp 60, COLL_P_HAp 50, and COLL_P.

Therefore, as shown by the microbiological analysis of the COLL_P scaffolds, HAp and marine collagen generate a synergistic antibacterial effect, resulting in enhanced antibacterial activity for the produced scaffolds [78]. Collagencin, an antimicrobial peptide identified in fish collagen, enhances the antibacterial properties compared with mammalian collagen [35].

With respect to the statistical analysis, the two-way ANOVA test indicated that the presence of 50:50 COLL:HAp in the formulation led to a statistical insignificant result (*p* > 0.05) compared to COLL_P against *P. aeruginosa*. For the same sample, the antibacterial effect was superior to COLL_P against *E. coli* and *S. aureus*, as indicated by the *p* value *p* < 0.05. Concerning the other two samples, with higher concentrations of HAp, the antibacterial effect was significantly increased compared to COLL_P for all tested bacteria (**** *p* < 0.0001).

With respect to microbial contamination, the purpose of this evaluation is to determine the microbial contamination of the COLL_P scaffolds according to the current edition of the European Pharmacopoeia 11th Edition [79]. Collagen scaffolds are considered non-sterile dosage forms. The microbial contamination assay, conducted on non-sterile perch collagen–HAp scaffolds, was performed by examining the level of microbial (bacterial and fungal) contamination and the presence or the absence of certain pathogenic microorganisms [80], namely *E. coli*, *S. aureus*, and *P. aeruginosa*.

The data presented in Table 3 comprise the total aerobic microbial count, TAMC, expressed as the mean colony-forming units (CFU) quantified on Soybean–Casein Digest Agar, and the total combined yeasts and filamentous fungi count, TYMC, expressed as the average CFUs determined on Sabouraud Dextrose Agar.

The microbial contamination assay results of the perch collagen–HAp samples from Table 3 show that TAMC values do not exceed the limits of admissibility provided by the European Pharmacopoeia, the current edition. COLL_P_HAp70 counts the smallest colonies of aerobic microorganisms, followed by COLL_P_HAp60, COLL_P_HAp50, and COLL_P, in accordance with the antibacterial activity. Regarding the TYMC values of the COLL_P scaffolds, no colony-forming units of yeast or mold were detected. Moreover, the scaffolds do not permit the growth of aerobic germs for any of the tested bacteria: *E. coli*, *S. aureus*, and *P. aeruginosa*.

The values found for TAMC, and the absence of combined yeast and molds, *E. coli*, *S. aureus*, and *P. aeruginosa*, permit us to affirm that the developed perch collagen–HAp scaffolds are suitable for potential use in bone tissue engineering.

### 3.8. Biocompatibility Evaluation

**Figure 13:**
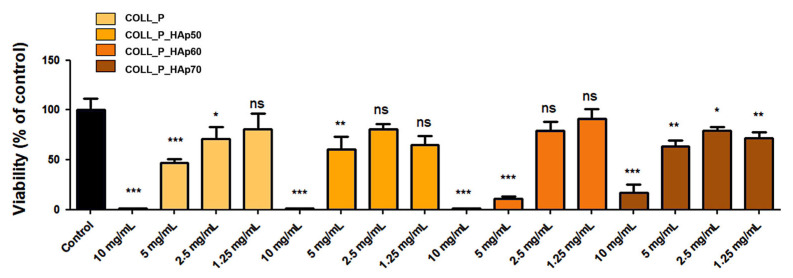
Viability of MG63 cells incubated for 72 h in the presence of extracts and their serial dilutions, as determined by XTT assay. The data is presented as mean ± standard deviation (* *p* < 0.5, ** *p* < 0.01, *** *p* < 0.001, ns, not significant).

The extracts of the tested scaffolds showed the cytotoxic effect at the 10 mg/mL concentration, which decreased for higher dilutions, as shown in Figure 13. This suggested the presence of soluble components (ions, degradation products, or residual reagents) that, in high concentrations, may disrupt cellular homeostasis, but whose effects became negligeable for most tested materials at 2.5 and 1.25 mg/mL. We could assume that, under physiological conditions, the scaffolds will possibly exhibit a favorable biocompatibility profile.

The next step was to assess the capacity of the tested scaffolds to support the growth and colonization with MG63 osteoblast-like cells.

**Figure 14:**
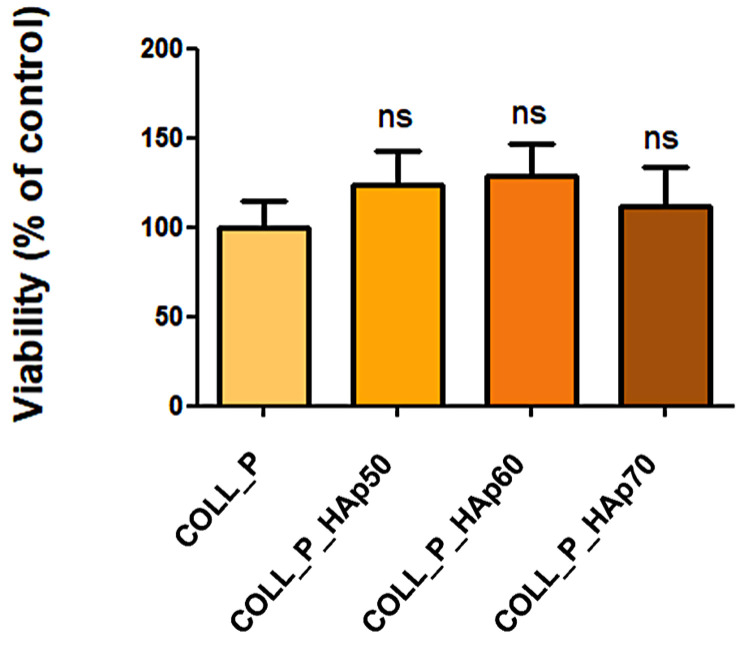
Viability of MG63 cells grown on the COLL_P and COLL_P_HAP scaffolds, after 1 week in culture, determined by XTT assay. The data is presented as mean ± standard deviation (ns, not significant).

All tested scaffolds supported MG63 cells viability, as shown in Figure 14, after one week of culture on the COLL_P and COLL_P_HAp materials. We can conclude that the addition of HAp did not impede cell attachment and survival on the biomaterials.

**Figure 15:**
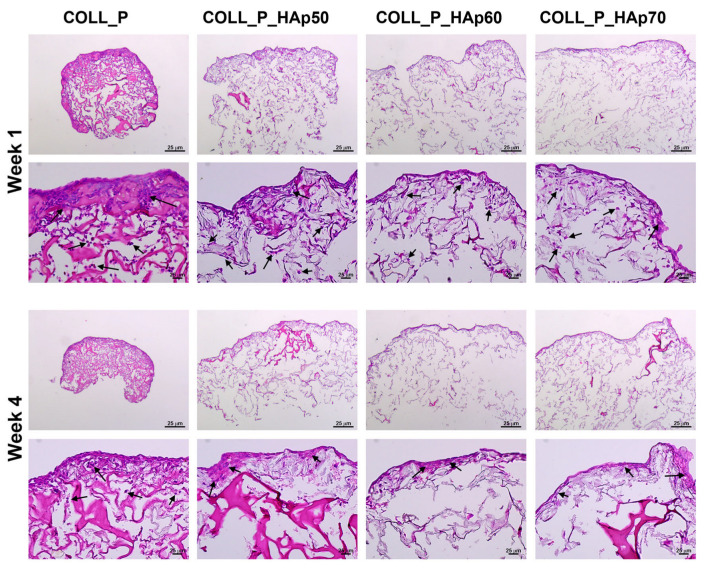
Haematoxylin and eosin staining illustrating the colonization of the COLL_P scaffolds and its derivatives with MG63 cells. Cells are indicated by black arrows.

Subsequently, we evaluated the capacity of collagen scaffolds to support colonization by MG63 cells. Hematoxylin and eosin staining showed that all scaffold types accommodated the three-dimensional growth of the MG63 osteoblasts one week after seeding, with cells present both on the surface and within the structural pores (Figure 15). These observations are consistent with the viability results shown in Figure 14. However, during the test, there was a notable contraction of the COLL_P sample, which, starting from the first week in culture, failed to preserve its architecture, a fact that is even more visible after four weeks. On the contrary, all the HAp-containing scaffolds preserved their three-dimensional structure in long-term culture, which is a key condition for biomaterials designed for bone regeneration, since the healing process of this hard tissue is weeks long, depending on the severity of the injury.

Nevertheless, there were differences regarding cell growth between scaffolds with various HAp percentages. As can be seen in Figure 15, in the lower panels (after fourweeks in culture), although for all these samples the cells remained mainly at the surface, lining the scaffold (probably due to the static cell culture conditions), and fewer cells were found inside the sponges, the best colonization can be observed in the case of COLL_P_HAp 50. For the other two samples, the cell presence became scarcer with increasing HAP percentage. Thus, we could conclude that, in the case of COLL_P_HAp 50, the proportion of the organic (collagen) and inorganic (HAp) components is optimal both for maintaining the three-dimensional structure for a longer period and for cell survival.

The results showed that a higher amount of HAp decreases biocompatibility; meanwhile, the microbiological results revealed that a higher amount of HAp improved the antimicrobial properties of sponges for all the tested strains: *E. coli*, *S. aureus*, and *P. aeruginosa*. This was expected to happen because the strong antimicrobials kill both microbes and cells and the balance between them is still a challenge [82].

At higher HAp ratios, the minor reduction in MG63 viability may be credited to several factors intrinsic to highly mineral-loaded composite scaffolds. One possible explanation is the increased local availability of Ca^2+^ and PO_4_^3−^ ions, which may transiently alter the ionic balance in the culture environment and influence early cellular responses. Additionally, higher HAp loading can modify surface microtopography, potentially affecting initial cell adhesion dynamics. As observed in Figure 14 and Figure 15, we did not observe a decrease in viability or colonization after one week in culture, suggesting that the initial adhesion was not affected by changes in surface microtopography or ion release. However, the decrease in colonization capacity observed in long-term culture could be attributed to these mechanisms. However, the present study was designed as a preliminary biological screening; thus, in depth mechanistic analyses such as ICP-OES ion-release profiling or live/dead fluorescence imaging were not included. Future investigations will address these facets to elucidate the interaction between scaffold mineral content, ionic release, and cell behavior.

This study has several limitations that should be considered. For example, the cytocompatibility assessment was performed using a cell line derived from osteosarcoma, MG63, which has osteoblast-like properties but does not fully replicate the behavior of primary human osteoblasts. On the other hand, the study focused on in vitro evaluation only and no in vivo experiments were performed to validate the regenerative potential of the scaffolds in a physiological environment. Future studies will address these aspects by incorporating primary bone cells, in vivo validation, and mechanical characterization to verify the clinical potential of the proposed scaffolds.

The scaffolds-based collagen and HAp presented in this research can be classified as a Class III implantable medical device according to Regulation EU 745/2017. This study is at the beginning stages but the best scaffold (COLL:HAp = 50:50) selected in this research could be the subject of preclinical studies, gamma sterilization, and scalability, and then clinical investigation and evaluation before obtaining a CE mark and going to market.

## 4. Conclusions

The aim of the present study was focused on developing novel scaffolds based on marine collagen and HAp, with potential application as advanced synthetic bone substitutes in bone tissue engineering. Structural analysis via FT-IR confirmed the main functional groups corresponding to the organic and inorganic phases, while SEM analysis revealed a structure with pores and a rough surface characteristic of a scaffold material that enhances cell proliferation. The porous structure of COLL_P scaffolds was confirmed by their swelling behavior. The collagen scaffolds supported colonization by MG63 osteoblasts, allowing the cells to grow in three dimensions, both on the surface and deep within the structural pores, one week after seeding. The COLL_P_HAp50 scaffold presents the optimal proportion between the organic (collagen) and inorganic (HAp) components, both for maintaining the three-dimensional structure for a longer period of time and for cell survival. The use of marine collagen extracted from perch skin enriched the potential antibacterial properties against *Escherichia coli*, *Staphylococcus aureus*, and *Pseudomonas aeruginosa*. Their biodegradability over time and the thermal properties of scaffolds were also investigated, proving that the perch collagen–HAp scaffolds developed are highly stable at high temperatures.

The study’s outcomes support the conclusion that the proposed scaffold represents a suitable and promising candidate for bone tissue engineering applications.

## Figures and Tables

**Table 1:** Composition of COLL_P samples.

Sample Name	COLL_P, %	Hap *, %	GA *, %
COLL_P	100	0	0.5
COLL_P_HAp50	50	50	0.5
COLL_P_HAp60	40	60	0.5
COLL_P_HAp70	30	70	0.5

* reported to collagen, dry substance.

**Table 2:** Thermogravimetric analysis of collagen related to each decomposition stage.

Sample	Mass Loss, %			Residual Mass, %	Tmax (DTG), °C
	25–100, °C	100–400, °C	400–700, °C		
COLL_P	5	46	16	33	315
COLL_P_HAp50	4	28	9	59	315
COLL_P_HAp60	3	23	7	67	315
COLL_P_HAp70	2	22	6	70	321

**Table 3:** Microbial contamination of perch collagen–HAp scaffolds.

Sample	TAMC * (CFU/g)	TYMC ** (CFU/g)	Detection of *E. coli*	Detection of *S. aureus*	Detection of *P. aeruginosa*
COLL_P	6.33	<1	Absent	Absent	Absent
COLL_P_HAp50	2.33	<1	Absent	Absent	Absent
COLL_P_HAp60	1.66	<1	Absent	Absent	Absent
COLL_P_HAp70	1.33	<1	Absent	Absent	Absent

According to pharmacopeia monographs, the allowance limits for TAMC are ≤1000 CFU/g and for TYMC are ≤100 CFU/g [79,81]. * TAMC is the total number of aerobic microorganisms. ** TYMC is the total combined yeast and molds count.

## Data Availability

Data is contained within the article.
